# Temperatures, diagnostics and treatment in pediatric cancer patients with fever in neutropenia, NCT01683370

**DOI:** 10.1038/s41597-020-0504-9

**Published:** 2020-05-26

**Authors:** Eva Brack, Stéphanie Wagner, Eveline Stutz-Grunder, Philipp K. A. Agyeman, Roland A. Ammann

**Affiliations:** 1 Division of Pediatric Hematology/Oncology, Department of Pediatrics, Inselspital, Bern University Hospital, Freiburgstrasse 15, 3010 Bern, Switzerland; 2 Division of Pediatric Nephrology, Department of Pediatrics, Inselspital, Bern University Hospital, Freiburgstrasse 15, 3010 Bern, Switzerland; 3 Department of Pediatric Oncology, University Children’s Hospital Zurich, Steinwiesstrasse 75, 8032 Zurich, Switzerland; 4 Department of Pediatrics, Inselspital, Bern University Hospital, Freiburgstrasse 15, 3010 Bern, Switzerland

**Keywords:** Paediatric research, Paediatric cancer

## Abstract

In pediatric oncology, there is no evidence-based definition of the temperature limit defining fever (TLDF), which itself is essential for the definition of fever in chemotherapy-induced severe neutropenia (FN). Lowering the TLDF can increase the number of FN episodes diagnosed. This prospective, single center observational study collected data on all temperature measurements, complete blood counts (CBCs), and measures of diagnostics and therapy performed at and after FN diagnosis in pediatric oncology patients using a high standard TLDF (39 °C ear temperature). In 45 FN episodes in 20 patients, 3391 temperature measurements and 318 CBCs, plus information on antibiotics, anti-fungal therapy, antipyretics, blood cultures taken and on discharge were collected. These data can mainly be used to study the influence of virtually lowering the TLDF on diagnostic measures, treatment and length of hospitalization in pediatric FN, which in turn are directly related to costs of FN therapy, and quality of life. This approach can be expanded to include as well different definitions of neutropenia.

## Abstract (summary)

**Table:** 

Measurement(s)	body temperature trait • Blood Cell Count • Diagnostics, Cancer • therapy
Technology Type(s)	Thermometer Device • complete blood cell count • Observational study
Factor Type(s)	day and time • outcome
Sample Characteristic - Organism	Homo sapiens
Sample Characteristic - Environment	hospital

Machine-accessible metadata file describing the reported data: 10.6084/m9.figshare.12118473

## Background & Summary

In children with cancer, fever in chemotherapy-induced neutropenia (FN) is the most frequent potentially lethal complication and the leading cause of emergency hospitalization^1^. Empirical therapy with broad-spectrum antibiotics, usually given intravenously in an in-house setting, are routine measures. They have led to a reduction of the FN associated mortality below 1% in developed countries^2^. However, there is no international consensus on temperature-limits defining fever (TLDF) for children with chemotherapy-induced severe neutropenia, despite its relevance for the clinical diagnosis and management of FN^1,3,4^. Published data on clinically used TLDF shows a wide range from 37.5 °C to 39.0 °C^5–7^. Through this inconsistency, the diagnosis of FN is made at varying temperatures. This reflects the fact that there is no internationally accepted evidence-based TLDF definition in pediatric fever in neutropenia^1^.

The TLDF applied determines not only the diagnosis of FN itself with initiation of therapy, but also the clinical measures of diagnostics and treatment during the whole hospitalization for FN. These measures include the number of blood cultures taken, the time-point of escalating empirical antibiotic therapy and the addition of empirical antifungal treatment in case of persisting fever^1^. Moreover, it influences the time point of hospital discharge and thus the length of hospitalization. These measures have a direct impact on the individual patient, including quality of life, and also on resource utilization and treatment costs^8^.

The here presented data^9^ has been collected during a prospective single-center study (August 2012 to May 2013), in pediatric cancer patients aged between ≥1 to ≤17 years undergoing chemotherapy, applying a historically established standard TLDF of 39.0 °C ear temperature (NCT01683370).

It is important to note that this study comprised two study questions in different clinical situations. The first situation was diagnosis of FN: Both the analytical results on the influence of virtually applying different TLDFs on FN diagnosis^7^ and the corresponding data descriptor^10^ have been published. The second situation was management of patients after FN diagnosis. The analytical results on the influence of virtually applying different TLDFs on this management have been published as well^11^. The corresponding data on diagnostic measures, treatment escalation and hospital discharge after FN diagnosis is presented here.

Our aim to publish these data that they can be merged with other data, to study (1) the differences of clinical management when applying different TLDF; (2) to develop clinical decision algorithms based on higher TLDF with the ultimate goal to (3) derive better resource utilization and a more cost-efficient management. Such merging may be performed, for example, with the detailed clinical data available in the recently published Paediatric Intensive Care database^12^.

During a cumulative duration of 289 months of chemotherapy, 45 FN episodes were diagnosed in 20 patients (maximum, 6 episodes per patient)^11^. Of these, 12 (27%) episodes had been diagnosed by the treating physician at temperatures below 39.0 °C for clinical reasons^7^.

**Fig. 1:**
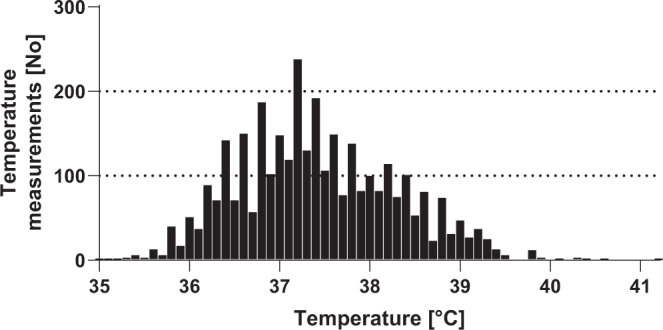
Temperature measurements in 45 FN episodes. Histogram depicting the 3391 temperatures measured 45 FN episodes.

During these 45 FN episodes, a total of 3391 temperatures were measured (median per episode, 56; IQR, 40 to 83; range, 7 to 401). Of these measurements, 193 were ≥39.0 °C (5.7%), and 937 ≥ 38.0 °C (27.6%) (Fig. 1). Furthermore, 318 (median per episode, 6; IQR, 5 to 7; range, 2 to 32) CBCs were performed. The ANC was ≤0.5 G/L in 208 (65%) of them.

In total, 122 (median per episode, 2; range, 1 to 11) BC were taken. Of these, 10 (8.2%) were reported positive, but 1 (0.8%) was considered false positive^11^. Thus in 9 of the 45 FN episodes (20%), bacteremia was detected. Intravenous antibiotics were given for a median of 5.7 days (range, 1 to 43).

For persisting fever ≥48 hours, i.v.-antibiotics were escalated in 25 (56%) episodes. Moreover, for persisting fever ≥120 hours, i.v.-antifungals were added in 4 (9%) episodes. The median length of stay was 5.7 days (range, 0.8 to 43.4).

These data can be used to simulate the effects of various TLDFs on the amount of diagnostic measures, on time-points and types of treatment escalation and on hospital discharge after FN diagnosis.

Regarding patients, there is an overlap of nearly 5 months (August 11, 2012, to December 31, 2012) with two separate data descriptors, both reporting retrospectively on patients under chemotherapy at risk to develop FN^13^, and on the corresponding FN episodes^14^ from January 1, 1993 to December 31, 2012. Except for temperatures that have led to FN diagnosis, there is no data overlap, however. Besides, the 45 temperature measurements and CBC leading to the diagnosis of FN in this study have been described before^10^.

## Methods

These methods are detailed versions of the methods published in our corresponding analytical work^11^.

### Study design

The data presented here have been collected during the prospective observational single center study “Pediatric FN Definition 2012 Bern” which was performed from August 2012 till May 2014 at the Division of Pediatric Hematology and Oncology, Department of Pediatrics, Inselspital, Bern University Hospital, University of Bern, Switzerland^9^. The study was conducted in accordance with the Declaration of Helsinki. The study was approved by the Institutional Review Board (Ethikkommission der Universitätskinderkliniken Bern) and the trial had been registered at www.clinicaltrials.com (NCT01683370) before starting patient accrual. Written informed consent was obtained from the patients, if able to judge, and from their legal guardians prior to study entry. This study was supported by an unrestricted research grant from the Swiss Cancer League (Grant No. KLS-2933-02-2012).

The division of Pediatric Oncology and Hematology in Bern covers a population of around one million inhabitants. The Division of Pediatric Hematology/Oncology unit treats around 40 newly diagnosed pediatric patients per year and has an outpatient unit as well as an inpatient unit with eight beds. Furthermore autologous stem cell transplantations for the majority of Switzerland, covering around 6 million inhabitants, are performed here. A high TLDF (39.0 °C tympanic temperature) is used clinically. The data described here were not published together with the corresponding analytical results, because they had to be fully anonymized before data sharing in order to comply with the current Swiss research legislation^7,11^.

### Patients and routine clinical management

All patients aged 1 to 17 years diagnosed with cancer and who were treated at the Department of Pediatrics, Inselspital, Bern University Hospital, requiring chemotherapy for ≥2 months at time of recruitment, were eligible. Upon withdrawal of informed consent or completion of chemotherapy (≥2 weeks after last dose and absolute neutrophil count (ANC) > 0.5 G/L), patients were off study. Of 40 potentially eligible patients, 39 participated in this study. Further information on patients’ characteristics has been published elsewhere^11^.

Patients were treated with chemotherapy, according to internationally established protocols. Except for standard *Pneumocystis jirovecii* prophylaxis, no primary antimicrobial prophylaxis was used^15,16^. Diagnosis of FN was made when a patient with severe chemotherapy-induced neutropenia developed fever. Fever was defined as a single temperature of ≥39.0 °C measured in the ear. Severe neutropenia was defined as an ANC ≤ 0.5 G/L or ≤1.0 G/L and expected to decline. If clinically indicated, the treating physician was free to diagnose FN at lower temperatures^2,17,18^.

### Management of fever in neutropenia

Routine clinical measures after FN diagnosis included emergency hospitalization and taking blood cultures (BC) from the central venous access device. Further details on clinical management have been published^2,18,19^.

After the diagnosis of FN was made, usually with TLDF of 39.0 °C, empirical antimicrobial treatment was started, usually ceftriaxone plus amikacin. For persisting fever despite treatment, the following clinical rules were applied: Additional BC every 24 h; escalation of the empirical intravenous antibiotics, usually to meropenem plus vancomycin after 48 hours; and addition of empirical intravenous antifungals, usually liposomal amphotericin B, after 120 hours^20^. Patients were discharged when clinically well and afebrile for ≥48 hours, and when they had negative blood cultures plus signs of bone marrow recovery.

### Data coverage of this study

This study collected data on temperature measurements, CBCs and diagnostic and therapeutic measures during FN episodes. Data on temperature and CBCs performed outside of FN are published elsewhere^10^. Data collected at the intersections, i.e., at FN diagnosis and at the end of the FN episode, are published in both datasets.

## Data Records

One single data record resulted from this trial, which contains a total of 45 single data files^9^, each for one specific FN episode. All the obtained data are published on Figshare: 10.6084/m9.figshare.5830236.v2.

## Technical Validation

### Reduction of recruitment bias

In order to reduce recruitment bias, all eligible patients from the cancer center were invited for study participation, regardless their diagnosis, age and gender. Only one patient denied study participation and so 39 of 40 eligible patients were recruited to participate. Thus, a relevant recruitment bias can be excluded. Furthermore, the participating study patients correspond to an unselected group of pediatric cancer patients regarding distribution of gender, age and diagnosis.

### Data collection and increasing data reliability

Within the setting of a prospective clinical study, the data presented here was extracted by an experienced pediatric oncology nurse from the clinical chart records into a commercially available spreadsheet program. A pediatric oncologist (RAA) checked the data for plausibility and, if not plausible, for agreement with the chart records.

A simple restricted FN definition was applied, based on verifiable quantitative information on both fever and neutropenia. The treating physician was free to diagnose FN below the standard TLDF of 39.0 °C ear temperature if clinically indicated.

All temperature measurements were performed using a single type of thermometer (Braun ThermoScan 5; IRT 4520; Braun GmbH, Kronberg, Germany; steps displayed, 0.1 °C; accuracy, ±0.2 °C; clinical repeatability, ±0.14 °C). CBC measurements were performed within clinical routine in a validated laboratory according to a standardized protocol.

### Anonymization procedure

Before publication, data were irreversibly anonymized using three processes in order to comply with Swiss research legislation. First, a random four-digit number, not back trackable and independent of the study patient identifier, was assigned to each patient. Second, data on diagnosis, age and gender, all considered to be potential identifiers, were deleted from the dataset. Third, the date of study start for each patient, as well considered a potential identifier, was replaced by day 0. Further days were correspondingly calculated from day 0 on. The real time of the day was expressed as a fraction of the day.

## Data Availability

No computer codes were used to generate the dataset. A commercially available spreadsheet program was used to collect the data and do quality checks.
